# Location-Specific Epigenetic Regulation of the Metallothionein 3 Gene in Esophageal Adenocarcinomas

**DOI:** 10.1371/journal.pone.0022009

**Published:** 2011-07-19

**Authors:** DunFa Peng, Tian-Ling Hu, Aixiang Jiang, Mary Kay Washington, Christopher A. Moskaluk, Regine Schneider-Stock, Wael El-Rifai

**Affiliations:** 1 Department of Surgery, Vanderbilt University Medical Center, Nashville, Tennessee, United States of America; 2 Department of Biostatistics, Vanderbilt University Medical Center, Nashville, Tennessee, United States of America; 3 Department of Pathology, Vanderbilt University Medical Center, Nashville, Tennessee, United States of America; 4 Department of Pathology, University of Virginia Health Center, Charlottesville, Virginia, United States of America; 5 Department of Pathology, Erlangen University, Erlangen Germany; 6 Department of Cancer Biology, Vanderbilt University Medical Center, Nashville, Tennessee, United States of America; Howard University, United States of America

## Abstract

**Background:**

Metallothionein 3 (MT3) maintains intracellular metal homeostasis and protects against reactive oxygen species (ROS)-induced DNA damage. In this study, we investigated the epigenetic alterations and gene expression of the MT3 gene in esophageal adenocarcinomas (EACs).

**Methods and Results:**

Using quantitative bisulfite pyrosequencing, we detected unique DNA methylation profiles in the MT3 promoter region. The CpG nucleotides from −372 to −306 from the transcription start site (TSS) were highly methylated in tumor (n = 64) and normal samples (n = 51), whereas CpG nucleotides closest to the TSS (−4 and +3) remained unmethylated in all normal and most tumor samples. Conversely, CpG nucleotides in two regions (from −139 to −49 and +296 to +344) were significantly hypermethylated in EACs as compared to normal samples [FDR<0.001, −log10(FDR)>3.0]. The DNA methylation levels from −127 to −8 CpG sites showed the strongest correlation with MT3 gene expression (r = −0.4, P<0.0001). Moreover, the DNA hypermethylation from −127 to −8 CpG sites significantly correlated with advanced tumor stages and lymph node metastasis (P = 0.005 and P = 0.0313, respectively). The ChIP analysis demonstrated a more repressive histone modification (H3K9me2) and less active histone modifications (H3K4me2, H3K9ace) in OE33 cells than in FLO-1 cells; concordant with the presence of higher DNA methylation levels and silencing of MT3 expression in OE33 as compared to FLO-1 cells. Treatment of OE33 cells with 5-Aza-deoxycitidine restored MT3 expression with demethylation of its promoter region and reversal of the histone modifications towards active histone marks.

**Conclusion:**

In summary, EACs are characterized by frequent epigenetic silencing of MT3. The choice of specific regions in the CpG island is a critical step in determining the functional role and prognostic value of DNA methylation in cancer cells.

## Introduction

Esophageal adenocarcinoma (EAC) is one of the human malignancies with the fastest growing incidence rates in the Western world [1], [2], [3]. It is generally considered that EAC develops from a premalignant lesion of Barrett's esophagus (BE) [3], [4]. BE is an acquired condition in which the normal squamous cell epithelium of the esophagus is replaced by a metaplastic columnar epithelium [5], [6]. Chronic gastro-esophageal reflux disease (GERD), with accumulation of reactive oxygen species (ROS) and subsequent oxidative DNA damage, is one of the main risk factors for the development of BE and its progression to adenocarcinoma [7], [8], [9], [10]. Hypermethylation of the gene promoter CpG islands is one of the major mechanisms to silence tumor suppressor genes and other tumor related genes [11], [12]. In addition to DNA methylation, other epigenetic alterations such as histone modifications, histone acetylation, and methylation are also involved in the regulation of gene expression [13], [14], [15]. Epigenetic cross talk between DNA methylation and histone modifications occurs through various physiologic and pathologic conditions. The combination of these dynamic interactions determines gene expression [16], [17].

Metallothioneins (MTs) are low in molecular weight (7 kDa) and are cysteine-rich proteins that are involved in maintaining intracellular metal homeostasis by binding transition metals such as zinc and copper [18], [19]. In mammalians, four members of the MT family have been identified [20]. The MTs play an important role in protecting against DNA damage, apoptosis and oxidative stress [20]. MT3 was discovered as an inhibitory neuronal growth factor that is involved in the reparative and/or protective processes in the brain and becomes down-regulated in Alzheimer's disease [21], [22]. The role of MT3 in tumorigenesis is unclear and the reported results were inconsistent. The levels of the MT3 protein are elevated in bladder [23], breast [24] and prostate cancers [25]; and this elevated expression was a poor prognostic indicator. Conversely, DNA methylation of the MT3 promoter has been associated with the down-regulation of the MT3 gene in gastric carcinoma [26] and esophageal squamous cell carcinoma [27]. Recent studies suggest that MT3 is involved in the protection of reactive oxygen species-induced DNA damage [28]. MT3 prevents the gamma-radiation-induced 8-oxoG accumulation and mutation in normal and hOGG1-depleted cells [29]. Because of MT3s antioxidant functions, we investigated the expression of MT3 and its epigenetic regulation in esophageal adenocarcinoma. Our results demonstrated epigenetic silencing of MT3 through promoter DNA hypermethylation and repressive histone modification mechanisms.

## Results

### Changes of DNA methylation levels in MT3 promoter region

**Figure 1:**
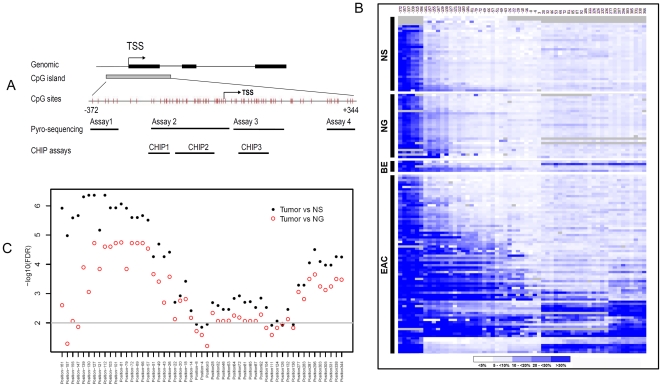
DNA methylation levels of the MT3 promoter in esophageal adenocarcinomas and normal tissue samples. **A**) Schematic illustration of MT3 genomic structure and the Pyrosequencing and ChIP assays. The MT3 gene comprises of 3 exons (black boxes). There is a long CpG island extending from upstream of the transcription start site (TSS) to the first intron (grey box). Each grey vertical bar represents one individual CpG site from −372 to +344. Four Pyrosequencing assays (assay 1–4) and three ChIP assays (ChIP 1–3) are shown. **B**) Heat map of DNA methylation profile from −372 to +344 in normal esophageal squamous mucosa (NS), normal gastric glandular mucosa (NG), Barrett's esophagus (BE) and esophageal adenocarcinoma (EAC) samples. A key for the blue scale is given below the map. Grey areas indicate missing data. **C**) Significance analysis of DNA methylation levels in the MT3 promoter region from −161 to +344 of TSS between EAC and normal samples. Each dot or cycle represents one comparison between tumor and normal samples for one CpG site, as indicated. The graph is plotted for the mean value in all tumor samples, as compared to the mean value for all normal samples (NS or NG) in each CpG site. FDR (false discovery rate) was used to demonstrate the significance and is plotted as −log10(FDR) in y axis. Thus, if FDR<0.01 then −log10(FDR)>2.0. We used FDR≤0.01 as the cutoff for statistical significance.

**Figure 2:**
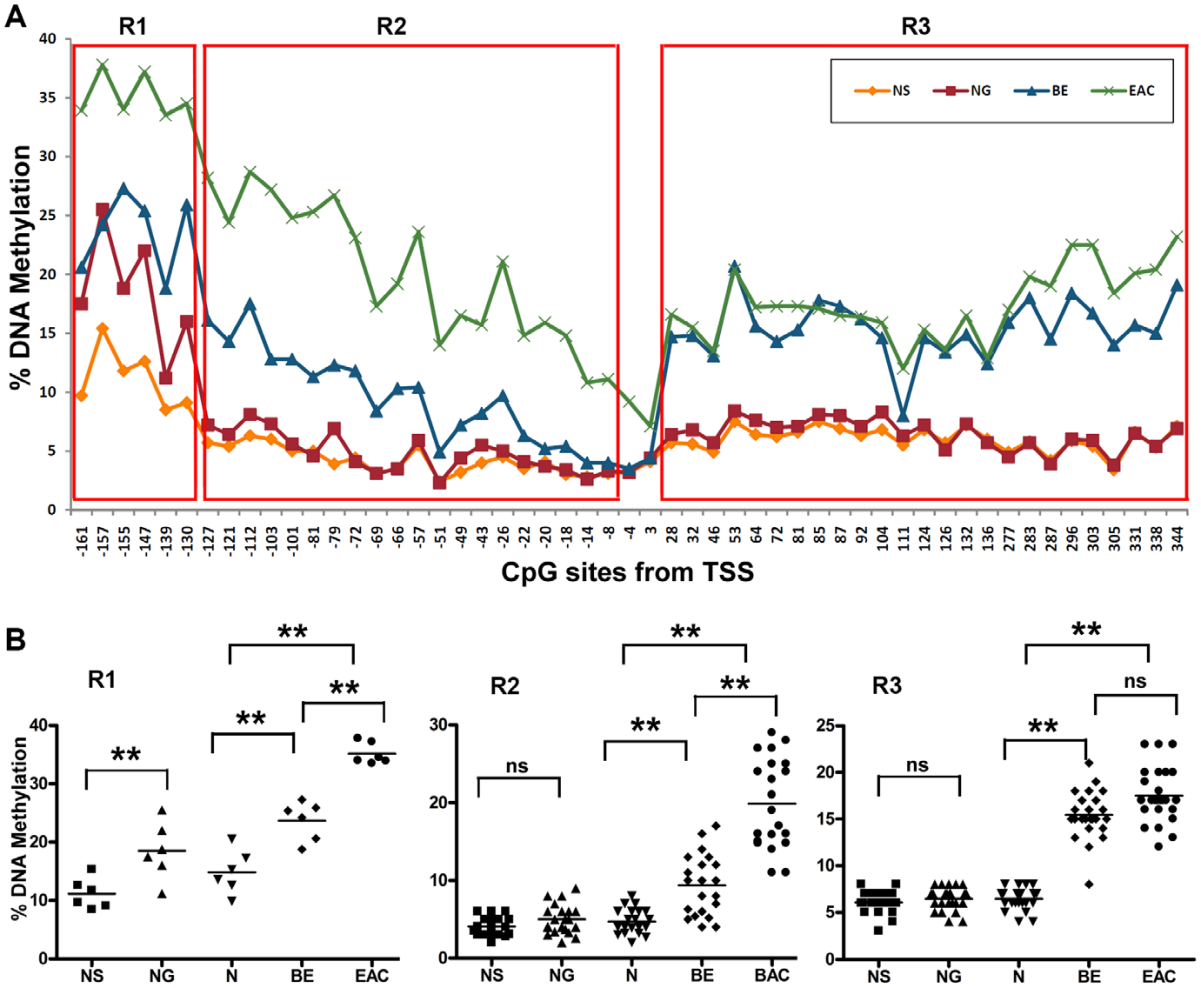
Biphasic changes of DNA methylation levels in MT3 promoter region from −160 to +344. **A**) The analysis included esophageal adenocarcinoma (EAC, n = 78), Barrett's esophagus (BE, n = 5), normal glandular gastric mucosa (NG, n = 28), and normal squamous esophageal mucosa (NS, n = 30). A biphasic DNA methylation change pattern was demonstrated in BE and EAC. According to the different DNA methylation patterns, we divided this region into 3 subregions (R1, R2 and R3) for further analysis. **B**) Statistical analysis of the DNA methylation level in R1, R2 and R3 in NS, NG, N, BE, and EAC. Each dot represents the average DNA methylation level of corresponding group samples of one CpG site. N is the mean of NS and NG. The horizontal bars in each group represent the mean methylation level. The difference of the DNA methylation levels between NS and NG were calculated with the Mann-Whitney test. The difference among N, BE and EAC and between pairs was calculated with ANOVA of Newman-Keuls test. ** indicates *P<0.01*, ns = not significant.

Our analysis indicated that the human MT3 gene contains a large CpG island located from −372 bp upstream of the transcript start site (TSS) to approximately +344 bp downstream of the TSS (Figure 1A). To determine the DNA methylation change of the CpG island, we designed several Pyrosequencing assays that enabled us to perform quantitative analysis of the DNA methylation level of each of the 59 CpG nucleotides using a state-of-the-art Pyrosequencing technique (Figure 1A). A heat map of the DNA methylation levels of each of the CpG sites from −372 to +344 of the TSS in NS (normal esophageal squamous epithelia), NG (normal gastric epithelia), BE, and EAC is shown in Figure 1B. A distant upstream region of CpG nucleotides from −372 to −306 displayed non-exclusive high level DNA methylation in all the normal and tumor samples (The 6 CpG sites at the left side in Figure 1B). This finding suggests that this region may not play a critical role in transcription regulation of MT3 during EAC carcinogenesis, and was excluded from further analysis. On the other hand, the normal samples (both NS and NG) displayed universal low levels of DNA methylation in the remaining CpG sites (Figure 1B), suggesting that the “field defect” phenomenon may not play a significant role in the regulation of MT3 gene expression. There was a significant difference in the DNA methylation level between EACs and both normal esophagus (NS) and normal stomach (NG) samples in almost all CpG nucleotides in the region from −161 to +344 (FDR<0.01, −log10(FDR)>2, Figure 1C). However, CpG nucleotides in two regions (−139 to −49 and +296 to +344) were more significant than all other sites (FDR<0.001, −log10(FDR)>3.0, Figure 1C). Interestingly, DNA methylation changes in this region displayed a biphasic methylation pattern in primary EACs: declining to <10% towards the TSS as seen in CpG nucleotides at −4 and +3 of TSS (Figure 2A). In our analysis, we divided the region from −161 to +344 into 3 subregions. The CpG nucleotides from −161 to −130 of the TSS (Region 1, R1) displayed increased DNA methylation in normal samples (10–25%). The methylation levels of these sites in normal stomach samples (NG) were significantly higher than in normal esophagus (NS, P<0.01). BE and EAC samples had higher DNA methylation levels than in normal samples (P<0.01 among N, BE, EAC, and between them, Figure 2B). The CpG nucleotides from −127 to −8 (Region 2, R2) demonstrated low methylation levels (below 10%) in both normal esophagus and stomach with a significant increase in the methylation levels in BE and EACs (P<0.001 among N, BE, EAC, Figure 2B). Similarly, the CpG nucleotides from +28 and +344 (Region 3, R3) showed low methylation levels in normal esophagus and stomach samples. However, this region showed similar higher methylation levels in BE and EACs, in particular, in sites from +28 to +277 (Figure 2A).

### DNA hypermethylation of the specific CpG nucleotides in EACs and correlation with down-regulation of MT3 gene expression

**Figure 3:**
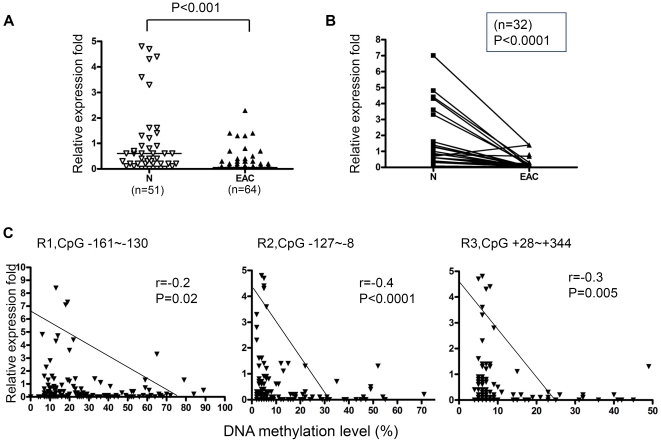
DNA methylation of the MT3 promoter correlates with MT3 gene expression. **A**) Comparison of MT3 gene expression between esophageal adenocarcinoma (EAC) and normal samples (N includes normal squamous esophageal mucosa (NS) and normal glandular gastric mucosa (NG)). **B**) MT3 gene expression in 32 matched normal (N) and tumor (EAC) pairs. **C**) The differential correlation power of Region 1 (R1, from −161 to −130), Region 2 (R2, from −127 to −8) and Region 3 (R3, from +28 to +344) was demonstrated. Spearman correlation analysis was used to evaluate the significance between MT3 DNA methylation and mRNA expression. The Mann-Whitney test was used in A and B analyses.

According to the unique DNA methylation changes shown above, the region from −161 to +344 in the MT3 promoter was considered to be important in esophageal adenocarcinogenesis. To explore the relative importance of the different subregions of this long promoter in regulating MT3 gene expression, 115 RNA samples from the same frozen tissues that were used for DNA methylation analysis were subjected to real-time RT-PCR analysis to examine the mRNA expression level of MT3. In accordance with the high level DNA methylation in primary EACs, we detected significantly down-regulated MT3 gene expression in EACs compared to normal esophagus and stomach samples (Figure 3A and 3B). We further compared the relative MT3 expression fold to the average DNA methylation level of the CpG nucleotides in Regions 1, 2, and 3 using a Spearman correlation analysis. As shown in Figure 3C, significant reverse correlation between MT3 DNA methylation and the MT3 mRNA expression were observed in R2 (from −127 to −8, r = −0.4, p<0.0001) and R3 (from +28 to +344, r = −0.3, p = 0.005). The strongest correlation was found to be in Region 2 where 63% (49/78) of EACs were hypermethylated (≥10% methylation level). Taken together, our results indicate that Region 2 (from −127 to −8) is the most suitable region for functional analysis of the MT3 promoter methylation; since it was frequently hypermethylated in EACs, had the strongest inverse correlation between DNA methylation and gene expression (Figure 3C), and was the best discriminating region between tumor and normal samples (Figure 1B).

### 5-Aza administration restores the expression of MT3

**Figure 4:**
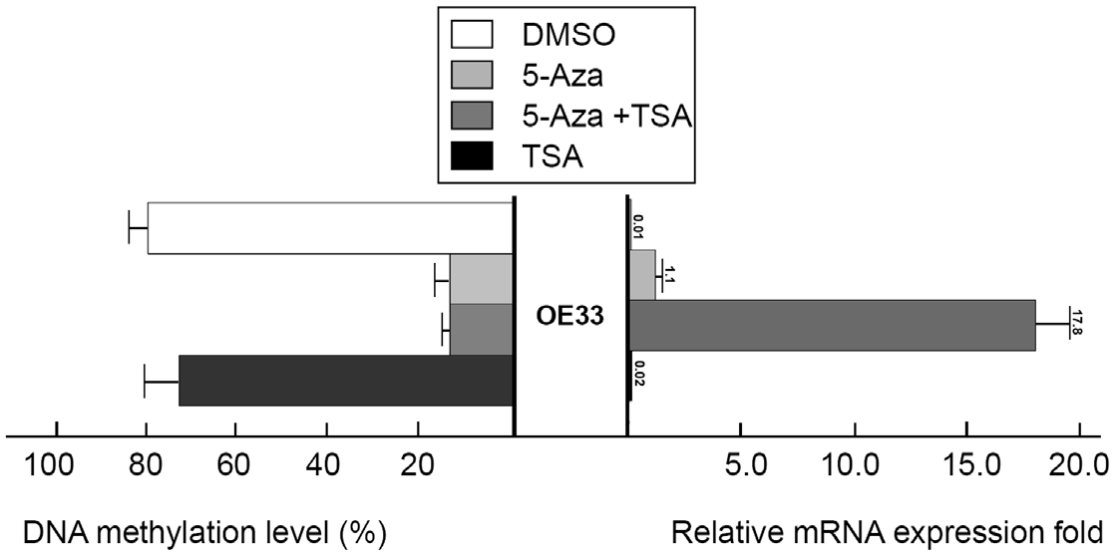
Treatment with 5-Aza and TSA reverses DNA methylation and gene expression patterns of MT3. An esophageal adenocarcinoma cell line (OE33) in which the MT3 gene is silenced and the MT3 promoter CpG island was highly methylated, were treated with 5-Aza and TSA as described in the Methods section. DNA methylation level was determined by Pyrosequencing in Region 2 (from −127 to −8). MT3 gene mRNA expression level was determined by real-time RT-PCR and normalized to HPRT to generate a relative fold induction. The 5-Aza treatment alone led to the induction of relative expression from 0.01 fold to 1.1 whereas the combination of 5-Aza and TSA led to an increase to 17.8.

To validate if DNA methylation is responsible for the gene silencing, an esophageal adenocarcinoma cell line (OE33) was treated with 5-Aza and TSA, as described in the Methods section. As shown in Figure 4, both 5-Aza alone and 5-Aza followed by TSA treatment restored MT3 gene expression in a previously silenced OE33 cell line accompanied by significant demethylation of a previously methylated promoter. TSA treatment alone did not have an effect on MT3 promoter methylation.

### DNA methylation of the MT3 gene promoter region associated with repressor histone modifications

**Figure 5:**
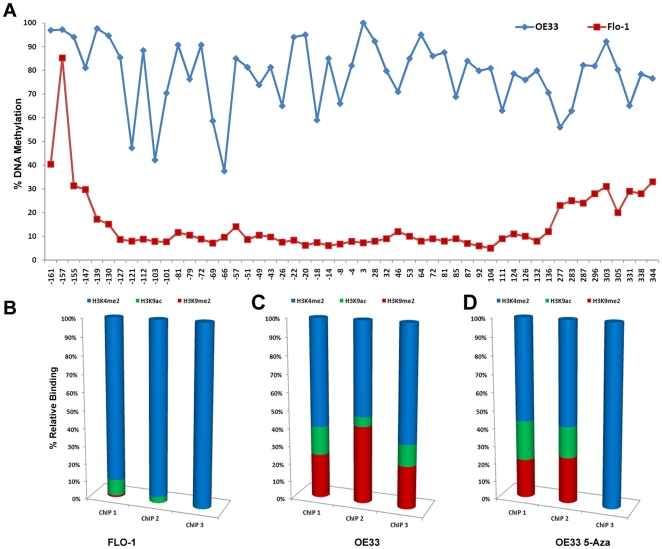
Quantitative Chromatin Immunoprecipitation (ChIP) assay of the MT3 promoter. The three ChIP assays of the MT3 promoter are shown in Figure 1A and Table 1. All ChIP experiments were performed in triplicates for each assay. Following quantitative real-time PCR (qPCR), the percent of relative of DNA binding in B, C and D was determined by normalizing the indicated histone binding level to its corresponding input and H3 levels. A relative percent of occupancy of three histones is shown. **A**) DNA methylation levels in the CpG nucleotides from −161 to +344 in OE33 and FLO-1 cell lines demonstrate a lower methylation level in FLO-1 cells. **B**) ChIP-qPCR in FLO-1 cells, demonstrate relatively low levels of repressive histone H3K9me2 as compared to histone H3K4me2 and H3K9ace. **C**) ChIP-qPCR results in the OE33 cell line show high levels of H3K9me2 as compared to the other two histones. **D**) ChIP-qPCR results in OE33 cell line, following administration of 5 µM 5-Aza for 72 h, reversed the relative abundance of the histone modifications towards active expression.

FLO-1 and OE33 cell lines were selected for the ChIP assays, based on their variable methylation and expression levels of MT3. OE33 cells have a high level of DNA methylation (74% for Region 2) and almost silenced MT3 expression, whereas FLO-1 cells express MT3 and have a low level of DNA methylation (9% for Region 2) (Figure 5A). To examine if the DNA hypermethylation of the MT3 gene promoter region is associated with additional repressor histone modifications, the chromatin immunoprecipitation assay (ChIP) was carried out using specific antibodies against H3, H3K4 dimethylation (H3K4me2), H3K9 dimethylation (H3K9me2), and H3K9 acetylation (H3K9ace). Three independent assays were developed along different regions of the MT3 promoter as shown in Figure 1A and Table 1. FLO-1 cells showed a very low level of repressive histone H3K9me2 as compared to histone H3K4me2 and H3K9ace (Figure 5B). In contrast, the OE33 cells displayed a significant level of H3K9me2 as compared to the other two histones (Figure 5C). Administration of 5-Aza (5 uM) to OE33 cells for 72 h reversed the relative abundance of the histone modifications towards active expression. There was a decrease in the level of H3K9me2 to MT3 promoter regions and an increase in H3K4ac and H3K4me2 (Figure 5D).

**Table 1:** Primers used for pyrosequencing, qRT-PCR, and ChIP analysis.

	Forward primer	Reverse primer	Sequencing primer
**Pyrosequencing**			
MT3-assay1	GGGTTAGGAGTTAAGGATTTGAAG	Bio-CTCCAAAACCCCAAATCTATTCA	TGAAGATGTTAGGTAGGATA (5s)
MT3-assay2	AGGTAGGGAAGAGTTGGGAAATA	Bio-CCACACACCCACTACATCTATC	CCACACACCCACTACATCTATC (2s); GGGGAGTGGGTTGGTA (1s); GATGAGGGGTGGGAG (3s)
MT3-assay3	AGGATGAGGGGTGGGAGT	Bio-CAACAAATAACCATACTTTTATAAC	GGTAGTGTATATATAAGGTAGG (4as); TAAGAGTATAAAAGGAAAGA (4ds); GAGTTTAGAGGGTTTGGTAT (4bs)
MT3-assay4	GATTTGTTTTTGTTTTTTTGGTGA	Bio-ACCCTTCCCCCAAATATCCTT	TTTGTTTTTTTGGTGAGT (7s)
**ChIP**			
ChIP1	GGAAGAGAGGCAGGGAAGAG	AGCGCGCACGTACCATCT	
ChIP2	TCTCCACTTTCGGAGATGGT	GCGTGCACGACCCACT	
ChIP3	AAGCGCACAAACGGAAAG	GTGGCGATGCTTTTATAGCC	
**qRT-PCR**			
MT3	GACATGGACCCTGAGACCTG	TCCTTGGCACACTTCTCACA	

### DNA methylation of the MT3 gene promoter region associated with advanced tumor stages

**Figure 6:**
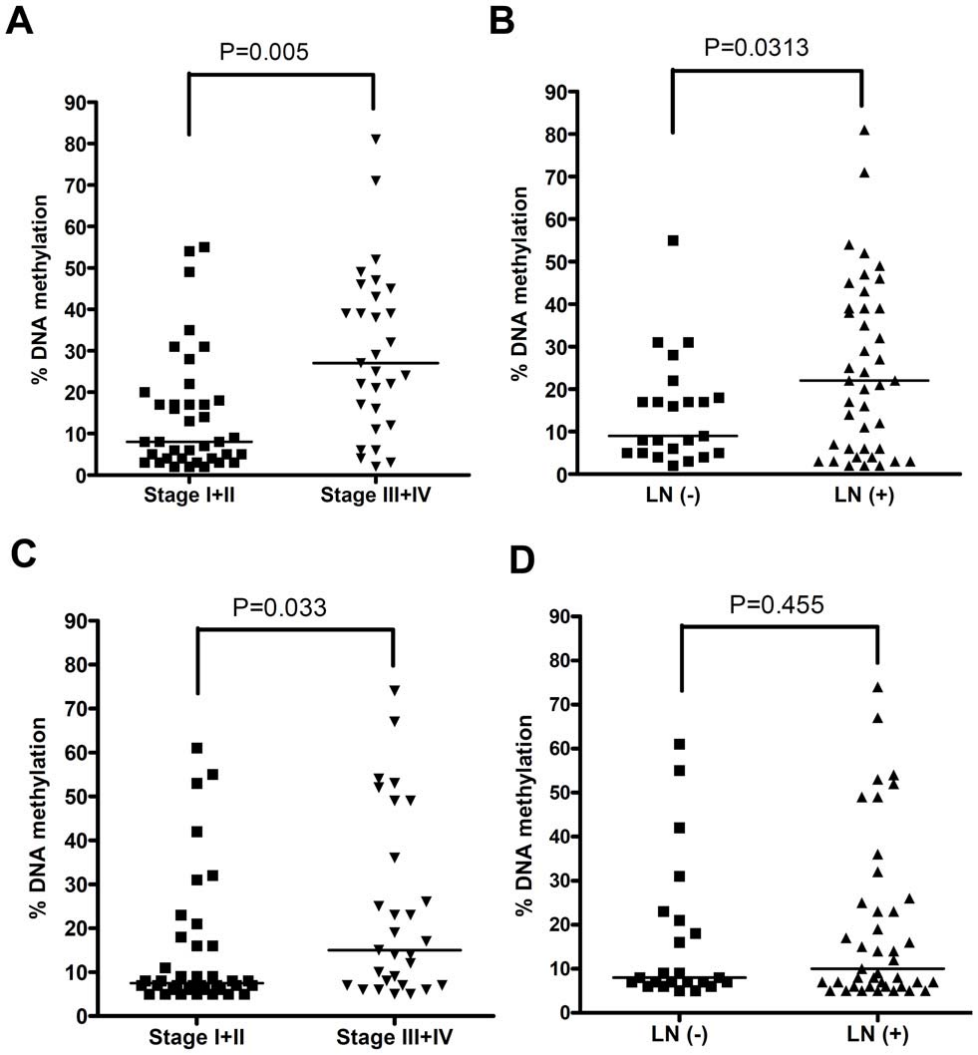
MT3 methylation of is associated with advanced tumor stage and lymph node metastasis. **A**) Increased DNA methylation of MT3 promoter R2 (−127 to −8 sites) *correlates with* advanced tumor staging (*P = 0.005*). **B**) DNA methylation of MT3 promoter R2 (−127 to −8 sites) *correlates with* lymph node metastasis (*P = 0.03*). **C**) DNA methylation of MT3 promoter R3 (+28 to +344 sites) *correlates with* tumor staging (*P = 0.03*). **D**) DNA methylation of MT3 promoter R3 (+28 to +344 sites) *does not correlate with* lymph node status (*P = 0.4*).

To investigate if MT3 promoter methylation is associated with tumor biology, we performed statistical analysis between DNA methylation and clinicopathological parameters. As shown in Figure 6, DNA methylation of R2 (from −127 to −8 CpG sites) was significantly associated with advanced tumor stages (*P = 0.005*) and lymph node metastasis (*P = 0.031*). However, DNA methylation R3 (from +28 to +344 sites) was only associated with tumor stage (*P = 0.033*) but not with lymph node metastasis (*P = 0.45*).

## Discussion

In this study, we demonstrated a unique DNA methylation pattern along the promoter CpG island of the MT3 gene. The MT3 CpG island demonstrated variable degrees of methylation. Interestingly, several CpG nucleotides were highly methylated in tumor and normal samples (from −372 to −306) whereas CpG nucleotides closest to the TSS (from −4 and +3) remained unmethylated in all normal and most tumor samples. On the other hand, CpG nucleotides in two regions (from −139 to −49 and +296 to +344) were more significantly hypermethylated in EACs as compared to normal samples (FDR<0.001, −log10(FDR)>3.0). The methylation pattern in BE was similar to that in EACs; however, BE had an extended region with unmethylated CpG nucleotides around the TSS (from −69 to +3). These results suggest the existence of some protective mechanism(s) that prevent the core region of the TSS from methylation in normal conditions [30], [31]. Although the mechanisms explaining progressive methylation patterns remain unclear, recent studies suggested that dimethylation of lysine 4 of histone 3 (H3K4me2) may protect against DNA methylation [32], [33]. Our ChIP results also support this hypothesis by showing a high level of H3K4me2 in an active MT3 promoter (in particular, in ChIP 2) in the FLO-1 cell line and a significantly decreased level of H3K4me2 in a suppressive promoter in OE33.

The unexpected variable methylation levels in the MT3 promoter CpG island highlight the critical importance of selecting the proper promoter sites for DNA methylation studies in cancer. Comprehensive analysis of the promoter regions using quantitative techniques such as Pyrosequencing may be important for identifying crucial promoter region(s) that regulate gene expression. This is best exemplified by our results in the region from −372 to −306, where DNA methylation was high in all samples including normal tissues without any correlation with the gene expression levels. On the other hand, the strongest inverse correlation between DNA methylation and gene expression was present in R2 (from −127 to −8), suggesting that this region may be critical in regulating MT3 gene expression. Significance analysis demonstrated that R2 has the smallest false discovery rate (FDR<0.001, −log10(FDR)>3) in differentiating EACs from both NS and NG. The difference between BE and normal tumors (NS+NG) and between EACs and BE were also statistically significant (p<0.01, respectively). Taken together, our findings suggest that R2 is the most suitable region to examine DNA methylation that possibly plays a critical role in transcription regulation of MT3. We, and others, have shown that DNA methylation could be an early event in carcinogenesis [34], [35], [36]. Our analysis of a few BE samples that were available for DNA methylation analysis suggested that R1 and R3 of the MT3 promoter may be early events occurring in BE. However, a study using a large panel of precancerous lesions, including Barrett's dysplasia samples, is needed to fully understand the progressive mode of MT3 methylation in the early stages of Barrett's carcinogenesis.

Using an *in vitro* cell model of EAC, we confirmed that DNA methylation is the mechanism underlying MT3 gene silencing. In a cell model of OE33, the treatment with 5-Aza demethylated the promoter region and restored MT3 gene expression, concordant with many other reports [35], [37]. Histone modifications including histone acetylation and methylation are important regulators of gene expression. Generally, active chromatin is associated with histone acetylation of H3K9 and methylation of H3K4, H3K36 and H3K79 while repressive chromatin is associated with histone deacetylating of H3K9 and methylation of H3K9, H3K27 and H4K20 [14], [38]. The final outcome of gene expression may be determined by the overall changes of these modifications, in addition to DNA methylation. We carried out ChIP assay followed by qPCR using two esophageal adenocarcinoma cell lines; FLO-1, which expresses MT3 and has a low level of DNA methylation; and OE33, which has hypermethylated promoter and silenced MT3. Analysis of three different regions of the MT3 promoter demonstrated significantly high levels of H3K4me2 and H3K9ace, and low levels of H3K9me2 in all three regions in FLO-1 cells. OE33 showed the converse results with an increased ratio of repressive to active histone H3K9 (H3K9me2/H3K9ace). The 5-Aza administration in the methylated OE33 cells reversed the ratio of repressive to active histone H3K9, showing higher levels of H3K9ace than H3K9me2. These results indicate that DNA methylation of the MT3 gene is linked with unique histone modifications in regulating gene expression. Surprisingly, the H3K4me2 level did not disappear completely, even in the highly methylated OE33 promoter. After 5-Aza treatment of OE33 cells, H3K4me2 showed a significant increase only in the ChIP3 region not in the ChIP2 region where the strongest correlation between DNA methylation and MT3 expression was observed. These results suggest that H3K4me2 may not be directly associated with MT3 gene expression. While some reports indicated that H3K4me2 is associated with active chromatin [39], [40] others demonstrated that H3K4me2 could be present on poised and inactive genes [14], [41]. The histone modifications and their relationship with DNA methylation and gene expression are quite complex [16], [38]. Further studies are needed to understand the complexity of epigenetic mechanisms in transcription regulation. Interestingly, we found that DNA methylation of the MT3 promoter region (R2, from −127 to −8 sites) was significantly associated with advanced tumor stages and lymph node metastasis (Figure 6), indicating that dysfunction of MT3 through DNA methylation of the core promoter region may be associated with tumor progression. These results further confirm the importance of selecting the appropriate promoter regions for the study of DNA methylation in cancer.

In conclusion, we demonstrated frequent silencing of MT3 expression in esophageal adenocarcinomas due to unique DNA methylation changes and histone modifications. The biological functions of MT3 in regulating ROS and oxidative stress in other diseases call for investigations along this line in esophageal carcinogenesis. The choice of a promoter region to examine DNA methylation is a critical step that should be carefully considered in molecular studies of carcinogenesis and in the development of early detection biomarkers.

## Materials and Methods

### Ethics Statement

De-identified human tissue samples were obtained from the archives of pathology at Vanderbilt University (Nashville, TN, USA), Erlangen University (Erlangen, Germany) and from the National Cancer Institute Cooperative Human Tissue Network (CHTN). The use of specimens was approved by the Institutional Review Board at Vanderbilt University Medical Center. All patients provided written consent, and samples were collected after surgical resection. All tissue samples that were included in this study were collected from tissues that remained after the completion of diagnosis, and are otherwise discarded.

### Cell lines

Esophageal adenocarcinoma cell lines, OE33 and FLO-1 [42], were cultured in Dulbecco's modified Eagle's medium (DMEM) media supplemented with 10% fetal bovine serum (FBS) and antibiotics (Invitrogen, Carlsbad, CA, USA) in a 37°C incubator with 5% CO_2_.

### Tissue samples

For DNA and mRNA analysis, 144 frozen tissue samples (78 EACs, 5 BE, 33 normal esophagus, and 28 normal stomach samples) were collected. The normal esophagus and stomach samples were taken from tumor-free margins of resected tumors and were histologically normal. Histopathological diagnosis of the BE and EAC was verified on the basis of H&E-stained sections, according to the Vienna classification of gastrointestinal epithelial neoplasia [43]. Fresh tissue samples were quickly rinsed in liquid nitrogen then embedded in optimal cutting temperature (OCT). A section was cut and H&E-stained. Tissue dissection was performed from OCT blocks, based on H&E staining, to obtain ≥70% tumor cell purity. All adenocarcinomas were classified according to the recent guidelines of the International Union Against Cancer (UICC) TNM classification system. All EACs originated from the lower esophagus or gastro-esophageal junction corresponding to the adenocarcinoma of the esophago-gastric junction [44]. The patients' ages ranged from 34–82 years (median at 63 years). The adenocarcinomas ranged from well differentiated to poorly differentiated, stages I–IV, with a mix of intestinal- and diffuse-type tumors.

### DNA bisulfite treatment and pyrosequencing analysis

DNA from cell lines and the primary tissue samples were purified using a DNeasy tissue kit (Qiagen, Valencia, CA, USA). The bisulfite modification of the DNA was performed using an EZ DNA Methylation-Gold Kit (ZYMO Research, Orange, CA, USA), according to the manufacturer's protocol. A 40 ng aliquot of modified DNA was subjected to PCR amplification of the specific promoter region containing a CpG island, by use of a primer set designed to amplify both methylated and unmethylated sequences of the MT3 gene. The primers were designed using PSQ assay design software (Qiagen), where one of the primers was biotin labeled. Several primer pairs were designed to sequence the whole promoter region from −372 to +344 (Figure 1A). The primers' sequences are given in Table 1. Platinum PCR SuperMix High Fidelity (Invitrogen) was used to prepare the PCR solution. The resultant PCR products were checked by gel electrophoresis to confirm the size of the product and rule out the formation of primer dimer. The specific PCR products were then subjected to quantitative pyrosequencing analysis using a Biotage PyroMark MD system (Qiagen) following the protocol provided by the manufacturer. The results were analyzed by Pyro Q-CpG 1.0.9 software (Qiagen). Based on control normal samples and internal quality controls provided in the software analysis, we used 10% as a cutoff for identification of DNA hypermethylation [35]. Statistical analysis was performed to detect significant changes in the frequencies of DNA methylation of CpG nucleotides between tumor and normal samples.

Based on the pyrosequencing data, we got the methylation level (%) of each CpG sites (from −372 to +344) from each individual sample including NS, NG, BE, and BAEs. We plotted these data into a heat map using xxx software.

### Quantitative real-time RT-PCR (qRT-PCR) analyses of MT3 expression

Total RNAs from all the cell lines and primary tissue samples were isolated using the RNeasy mini kit (Qiagen), and single-stranded cDNA was subsequently synthesized using the iScript cDNA synthesis kit (Bio-Rad, Hercules, CA, USA). The primers were designed using the online software, Primer 3 (http://frodo.wi.mit.edu/cgi-bin/primer3/primer3_www.cgi). The forward and reverse primers were designed to span two different exons. The primers' sequences are given in Table 1. The oligos were obtained from Integrated DNA Technologies (Coralville, IA, USA). The qRT-PCR was performed using an iCycler (Bio-Rad) with the threshold cycle number determined by use of iCycler software version 3.0. Reactions were performed in triplicate, and the threshold numbers were averaged. The results of these genes were normalized to *HPRT1*, which had minimal variation in all normal and tumor samples tested [45]. Expression fold was calculated according to the formula 2^(*Rt–Et*)^/2^(*Rn–En*)^ [45], where *Rt* is the threshold cycle number for the reference gene observed in the tumor, *Et* is the threshold cycle number for the experimental gene observed in the tumor, *Rn* is the threshold cycle number for the reference gene observed in the normal samples, and *En* is the threshold cycle number for the experimental gene observed in the normal samples. *Rn* and *En* values were calculated as an average of 30 normal samples.

### 5-Aza-2′-deoxycytidine (5-Aza) and Trichostatin-A (TSA) treatment

For validation of the role of DNA methylation in transcriptional regulation *in vitro*, an esophageal cancer cell line (OE33) was maintained in Dulbecco's modified Eagle's medium (DMEM), supplemented with 10% fetal bovine serum (FBS) and antibiotics (Invitrogen). Cells were seeded at low density for 24 h, and then treated with 5 µM 5-Aza (Sigma-Aldrich, St Louis, MO, USA) for 72 h and/or 200 nM TSA (Wako, Osaka, Japan) for 24 h. Total RNA and DNA were isolated and purified by the RNeasy kit and the DNeasy tissue kit (Qiagen) as described above. DNA methylation levels of MT3 in the samples before and after treatments were determined by pyrosequencing, and mRNA expression levels were determined by qRT-PCR as described above. For ChIP assay, chromatins from OE33 cells (after 5-Aza administration) were harvested following the protocol described in the section of chromatin immunoprecipitation (ChIP) assay.

### Chromatin immunoprecipitation (ChIP) assay

To check the role of histone modifications in the MT3 promoter region, chromatin immunoprecipitation (ChIP) assay was carried out [46]. In brief, chromatin in confluent cells of OE33 and FLO-1 lines was cross-linked and harvested, and the protein-chromatin lysate was fragmented to a length between 200 and 1,000 bp by sonication. The chromatin was precleaned by incubating with protein G agarose and then immunoprecipitated, with rotation, overnight at 4°C with the antibodies against histone 3 (H3), anti-acetylated histone H3K9 (H3K9ace, #9671, 1∶50, Cell Signaling, Danvers, MA, USA), dimethylated histone H3K4 (H3K4me2, #9725, 1∶50, C64G9, Cell Signaling), dimethylated histone H3K9 (H3K9me2, #9753, 1∶25, Cell Signaling). The precipitated chromatin was then harvested by protein G agarose and eluted in elution buffer after a series of washing. A ChIP DNA clean & concentration kit (ZYMO Research) was used on the chromatin following the manufacturer's instructions. The ChIP DNA was eluted in a final 50 µl volume, and 2 µL of each ChIP DNA was subjected to quantitative analysis by real-time PCR (qPCR). Primers were designed to amplify 3 regions in the MT3 promoter area as shown in Figure 1A. The primers' sequences are given in Table 1. Fold enrichment in the binding of histones (H3K4me2, H3K9ace, and H3K9me2) to MT3 promoter regions in each immunoprecipitation was normalized to the indicated histone binding level in its corresponding input and H3 levels in input DNA. Two independent ChIP experiments were performed for each analysis, and qPCR was performed twice for each of the ChIP DNA samples. The standard deviation among the experiments did not exceed 10% of the average values. The average values of the three histones were graphed to demonstrate the relative percent of occupancy.

### Statistical analysis

The Mann-Whitney test was used 1) to compare the DNA methylation level between NS and NG, and 2) to compare the mRNA expression fold between normal and tumor samples. ANOVA of Newman-Keuls test was used to compare the differences of the average DNA methylation level among N, BE and EAC. The significance in the DNA methylation level of each individual CpG site between EAC and normal samples was calculated using the Tukey method and expressed as false discovery rate (FDR) [47], then plotted as −log10(FDR). The correlations between the DNA methylation level and mRNA expression fold were determined by Spearman Correlation. All *p*-values were based on two-sided tests and differences were considered statistically significant when *p*-value≤0.01.
